# Author Correction: Discovery of an exosite on the SOCS2-SH2 domain that enhances SH2 binding to phosphorylated ligands

**DOI:** 10.1038/s41467-023-42706-4

**Published:** 2023-12-01

**Authors:** Edmond M. Linossi, Kunlun Li, Gianluca Veggiani, Cyrus Tan, Farhad Dehkhoda, Colin Hockings, Dale J. Calleja, Narelle Keating, Rebecca Feltham, Andrew J. Brooks, Shawn S. Li, Sachdev S. Sidhu, Jeffrey J. Babon, Nadia J. Kershaw, Sandra E. Nicholson

**Affiliations:** 1 The Walter and Eliza Hall Institute of Medical Research, Parkville, VIC Australia; 2 Department of Medical Biology, University of Melbourne, Parkville, VIC Australia; 3 The Donnelly Center for Cellular and Biomolecular Research, University of Toronto, Toronto, ON Canada; 4 The University of Queensland Diamantina Institute, Woolloongabba, QLD 4102 Australia; 5 Department of Biochemistry and the Siebens-Drake Medical Research Institute, Schulich School of Medicine and Dentistry, University of Western Ontario, London, ON Canada

**Keywords:** Cell signalling, Peptides, X-ray crystallography, Phosphorylation

Correction to: *Nature Communications* 10.1038/s41467-021-26983-5, published online 02 December 2021

The original version of this article contained an error in Table 2, in which the original bottom 5 rows showed the incorrect values. The correct version of Table 2 is:

**Table:** 

	Location	GHR pY595 (μM)	F3 (μM)	GHR pY595 + F3 (μM)*
**SOCS2 mutation**				
WT		1.08 ± 0.07	0.67 ± 0.03	0.15 ± 0.02
L36A	ESS	0.80 ± 0.02	ND^1^	0.50 ± 0.02
L40F		1.03 ± 0.07	0.81 ± 0.02	0.29 ± 0.02
L40A		1.76 ± 0.21	>50	0.46 ± 0.08
R41A		1.18 ± 0.54	0.89 ± 0.05	0.25 ± 0.07
Q45A		1.32 ± 0.03	0.60 ± 0.34	0.22 ± 0.03
Y49F	*β*A	0.98 ± 0.01	0.79 ± 0.01	0.18 ± 0.01
Y99A	*β*D	2.03 ± 0.05	ND	1.84 ± 0.4
L81F	*β*C	1.88 ± 0.01	ND	1.75 ± 0.27
L82F	*β*C	1.17 ± 0.04	1.53 ± 0.03	0.45 ± 0.02
L81, 82F	*β*C	0.93 ± 0.34	ND	1.94 ± 0.27

which replaces the previous incorrect version:

**Table:** 

	Location	GHR pY595 (μM)	F3 (μM)	GHR pY595 + F3 (μM)*
**SOCS2 mutation**				
WT		1.08 ± 0.07	0.67 ± 0.03	0.15 ± 0.02
L36A	ESS	0.80 ± 0.02	ND^1^	0.50 ± 0.02
L40F		1.03 ± 0.07	0.81 ± 0.02	0.29 ± 0.02
L40A		1.76 ± 0.21	>50	0.46 ± 0.08
R41A		1.18 ± 0.54	0.89 ± 0.05	0.25 ± 0.07
Q45A		1.32 ± 0.03	0.60 ± 0.34	0.22 ± 0.03
Y49F	*β*A	0.98 ± 0.01	0.79 ± 0.01	0.18 ± 0.01
S78A	BC loop	2.03 ± 0.05	ND	1.84 ± 0.4
Y99A	*β*D	1.88 ± 0.01	ND	1.75 ± 0.27
L81F	*β*C	1.17 ± 0.04	1.53 ± 0.03	0.45 ± 0.02
L82F	*β*C	0.93 ± 0.34	ND	1.94 ± 0.27
L81, 82F	*β*C	1.08 ± 0.07	0.67 ± 0.03	0.15 ± 0.02

This has been corrected in both the PDF and HTML versions of the Article.

