# Supplementary material for: Discovery of an exosite on the SOCS2-SH2 domain that enhances SH2 binding to phosphorylated ligands
Source: Nat Commun. 2021 Dec 2;12:7032. doi: 10.1038/s41467-021-26983-5 (PMC8640019; doi:10.1038/s41467-021-26983-5)
Supplement: Supplementary file 1 — Supplementary Information [file 41467_2021_26983_MOESM1_ESM.pdf]

Supplementary Information

Discovery of an exosite on the SOCS2-SH2 domain that enhances SH2 binding to phosphorylated ligands

Edmond M. Linossi[1,2]*, Kunlun Li[1,2]*, Gianluca Veggiani[3]*, Cyrus Tan[1,2], Farhad Dehkhoda[1,2], Colin Hockings[1,2], Dale Calleja[1,2], Narelle Keating[1,2], Rebecca Feltham[1,2], Andrew J. Brooks[4], Shawn S. Li[5], Sachdev S. Sidhu[3], Jeffrey J. Babon[1,2], Nadia J. Kershaw[1,2]†, Sandra E. Nicholson[1,2]†

[1]The Walter and Eliza Hall Institute of Medical Research, Parkville, Australia;

[2]Department of Medical Biology, University of Melbourne, Parkville, Australia;

[3]The Donnelly Center for Cellular and Biomolecular Research, University of Toronto, Toronto, Canada;

[4]The University of Queensland Diamantina Institute, The University of Queensland, Translational Research Institute, Woolloongabba, QLD 4102, Australia

[5]Department of Biochemistry and the Siebens-Drake Medical Research Institute, Schulich School of Medicine and Dentistry, University of Western Ontario, London, Canada.

* These authors contributed equally to this work.

† These authors jointly supervised this work and are corresponding authors.

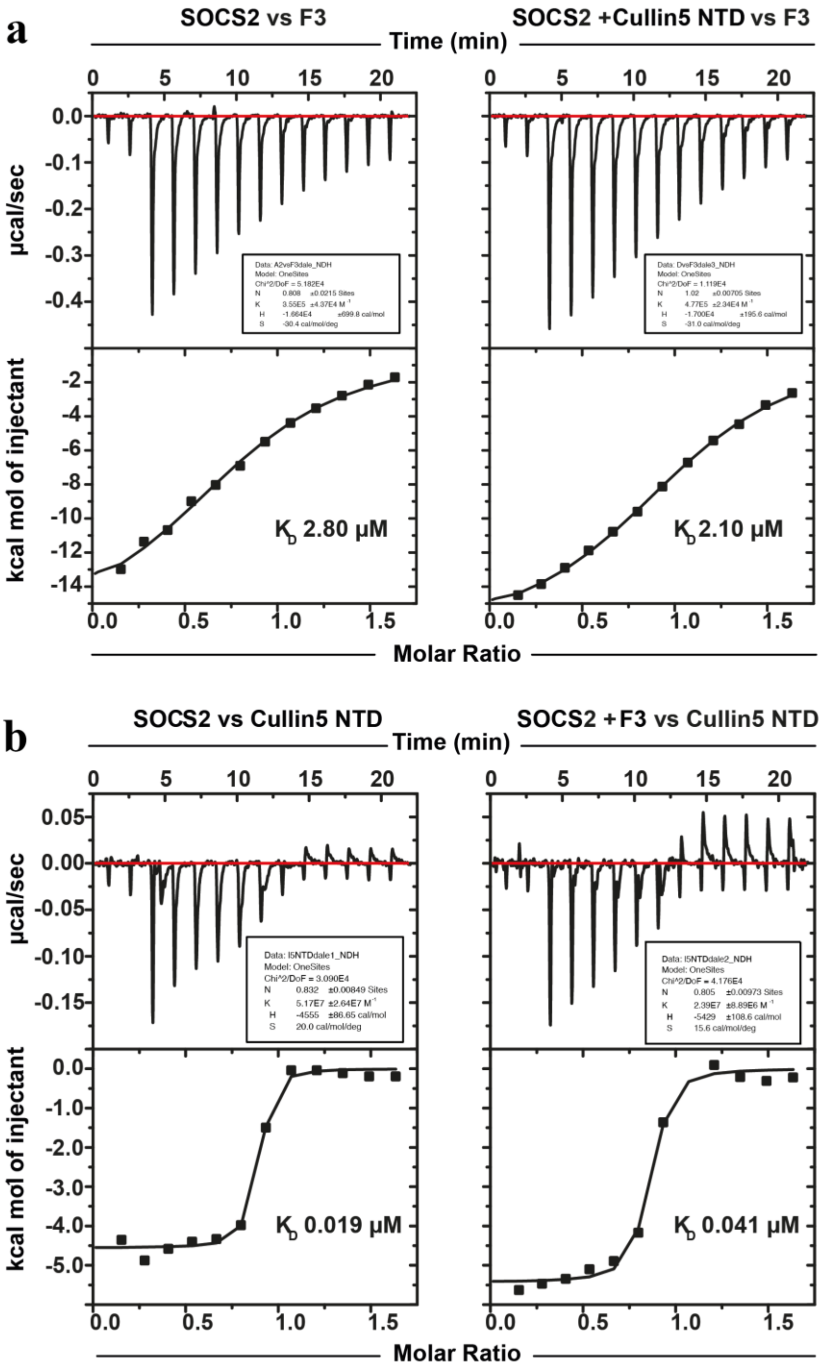

Supplementary Figure 1. F3 binding to SOCS2 is independent of SOCS-box interaction with Cullin5. (a) Representative ITC curves showing that SOCS2 interaction with the non-phosphorylated peptide F3 (left hand side), is not disrupted by Cullin5 N-terminal domain (NTD) binding (right hand side). (b) Representative ITC curves showing that binding of Cullin5 NTD (left hand side) is not disrupted by F3 binding (right hand side). K_D values are the average of two independent experiments. Additional details in Supplementary Table 2.

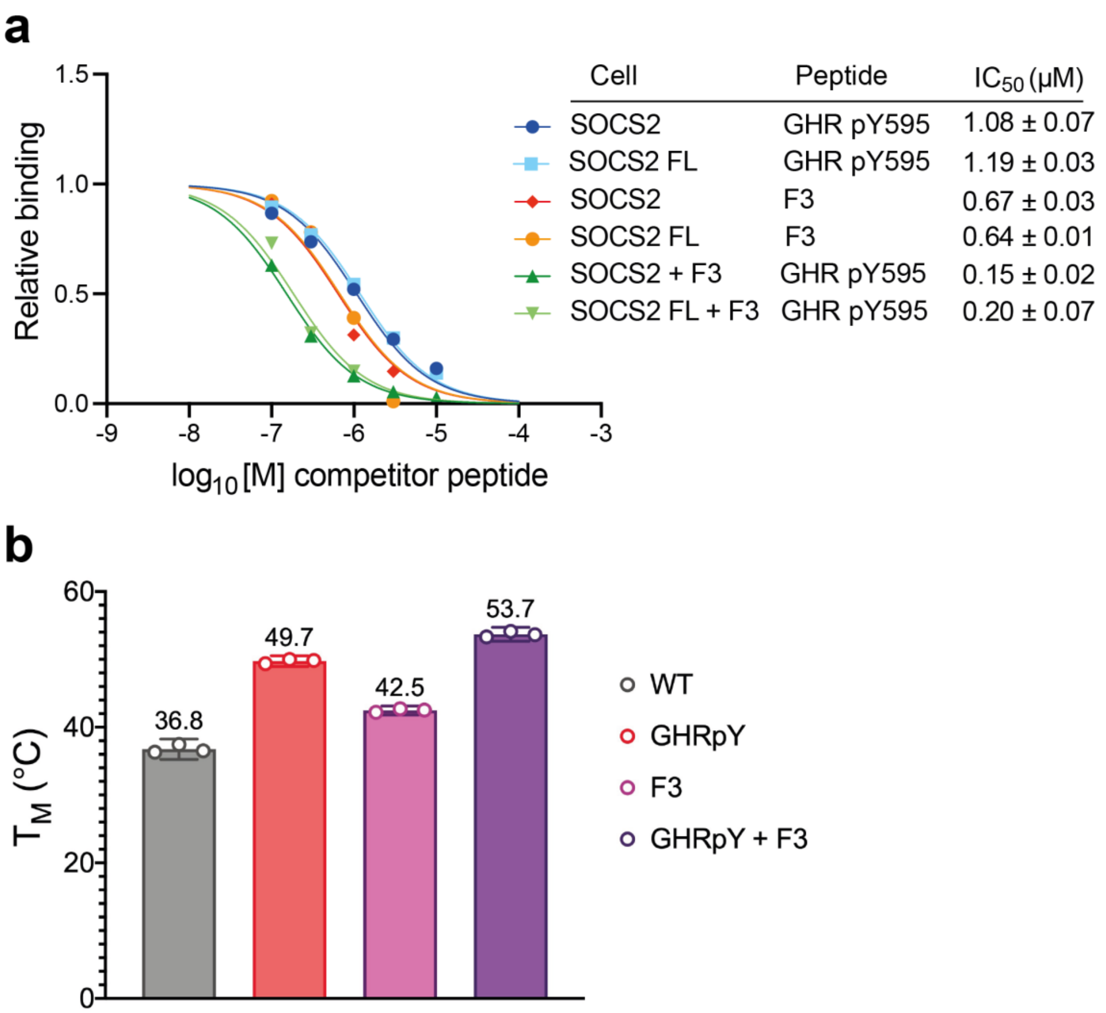

Supplementary Figure 2. F3 binds with similar affinity to both SOCS2 lacking the N-terminal region and full-length SOCS2, and is sufficient to both enhance binding to a phosphorylated ligand and confer thermostability. (a) Representative SPR curves indicate $SOCS2^{32\text{-}198}$-EloB/C and full-length (FL) SOCS2 ($SOCS2^{1\text{-}198}$-EloB/C) display comparable binding to F3 and show similar F3 enhancement of GHR pY595 peptide (GHR) binding. Values are mean ± S.D. and are derived from n=3 independent experiments. (b) SOCS2-SH2 domain alone ($SOCS2^{32\text{-}159}$) was incubated with either 50 µM GHR pY595 peptide, 50 µM F3 peptide, or both peptides in HEPES-buffered saline for 5 min before addition of Sypro-Orange and heating in a Thermal Cycler. Melting temperatures were obtained by performing a Boltzmann sigmoidal non-linear fit of normalised data. Values and error bars represent mean and 95% confidence interval from the non-linear fit of technical triplicates, and are representative of 3 independent experiments. Source data are provided as a Source Data file.

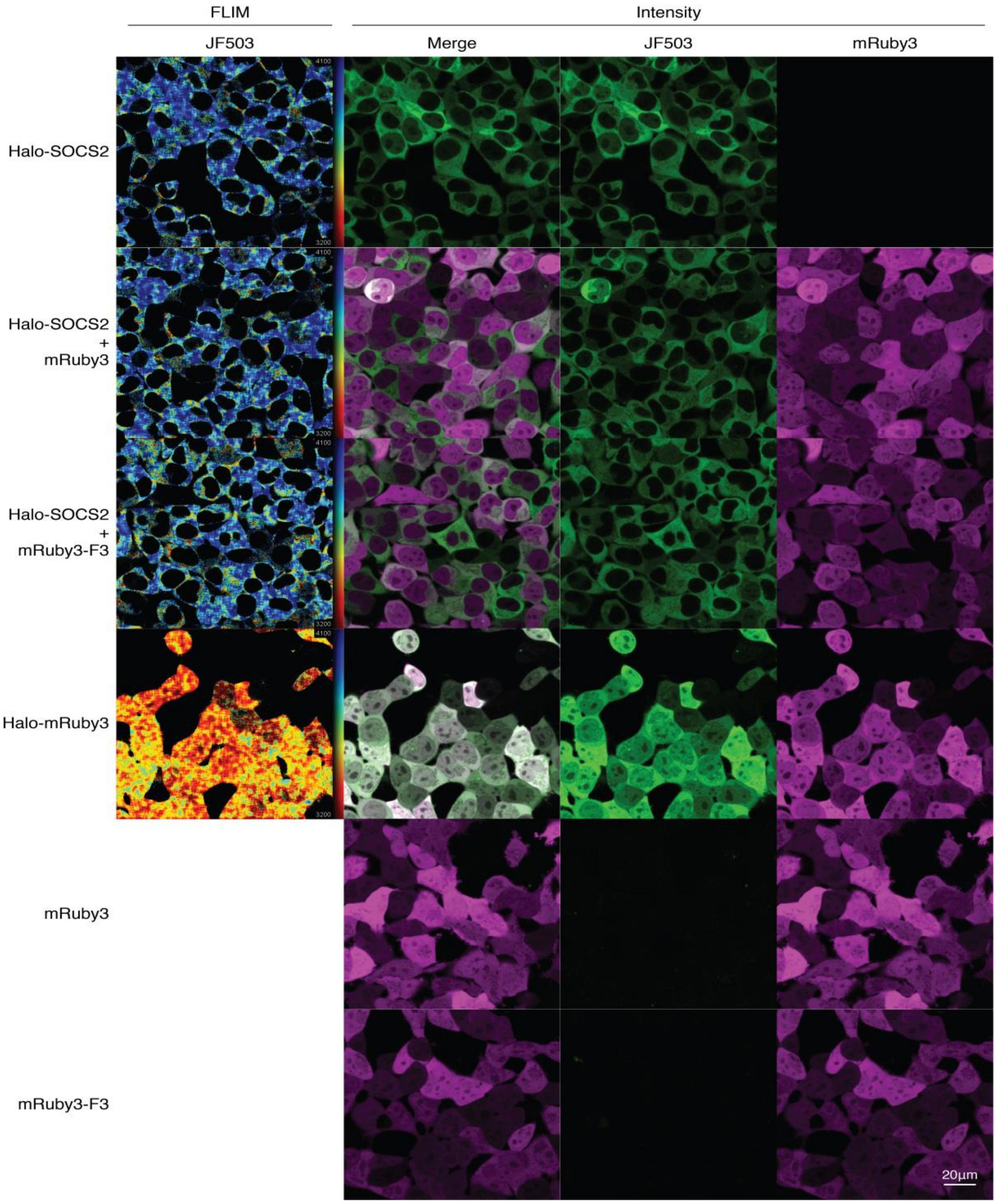

Supplementary Figure 3. Halo-SOCS2 and F3 interact in live cells. SOCS2 labeled with JF503 showed a specific FRET interaction with mRuby3-tagged F3. HEK293T cells expressing Halo-SOCS2 (dox-inducible) and/or stably expressing mRuby3-F3 (or mRuby3 alone, or the positive control Halo-mRuby3), as indicated, were stained with Halo ligand JF503 and imaged by fluorescence lifetime imaging microscopy (FLIM). Representative images show lower fluorescence lifetimes (rainbow colour scheme skewed more red/yellow) in cells expressing SOCS2 and mRuby3-F3 than cells expressing only SOCS2, or SOCS2 and mRuby3 without F3. The positive control Halo-mRuby3 represents maximum FRET interaction, as the JF503 and mRuby3 fluorophores are physically linked together**.** Images displayed are identical with respect to instrumentation, analysis and display settings, and are representative of 3 independent experiments. FLIM images are displayed with a rainbow colour scale, with lower and upper limits of 3200 ps and 4100 ps (lifetime), respectively.

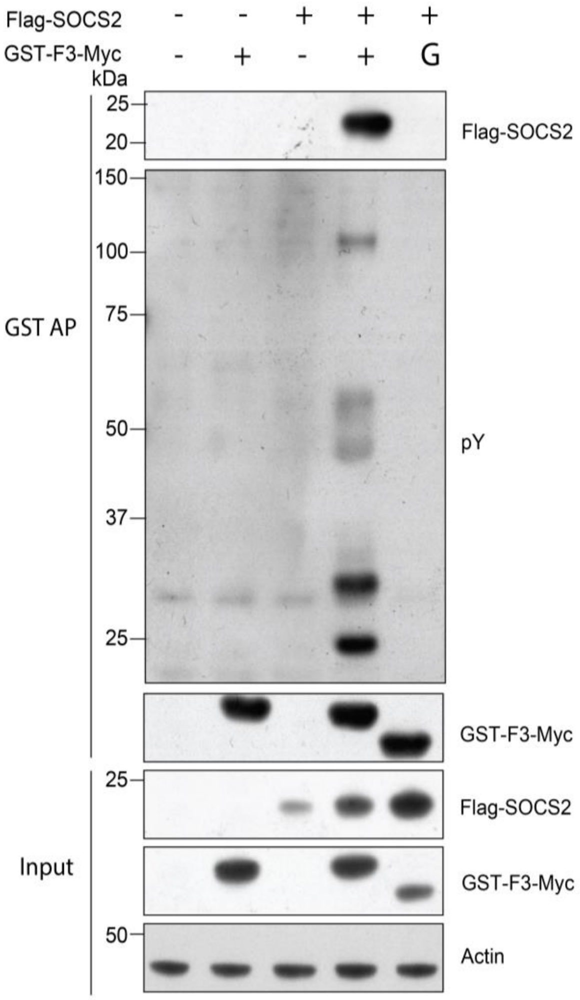

Supplementary Figure 4. F3 binding to SOCS2 enhances enrichment of tyrosine phosphorylated proteins (pY). 293T-GHR cells were transfected with constructs expressing GST-F3-Myc and/or Flag-tagged SOCS2, treated with 50 ng/mL GH for 30 min and lysed, prior to enrichment of GST-F3-Myc protein complexes with glutathione-Sepharose. As a control, cells were transfected with a GST-Myc construct (G). Enriched proteins were analyzed by immunoblotting with the indicated antibodies. GST-Myc constructs were blotted with anti-Myc antibodies. Representative of 2 independent experiments. AP: affinity precipitation. Source data are provided as a Source Data file.

a

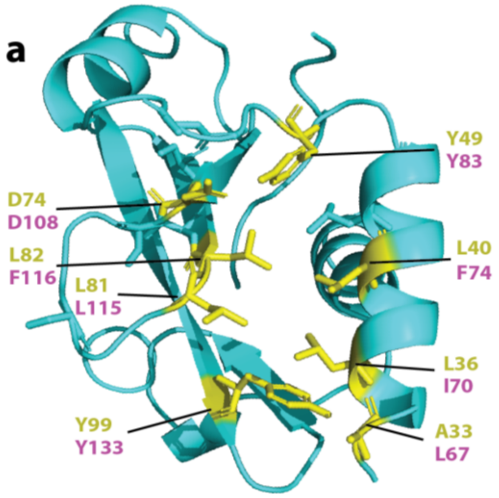

b

CIS	pY595 IC$_{50}$ (μM)	F3 IC$_{50}$ (μM)	+ F3 pY595 IC$_{50}$ (μM)
**Wild-type**	15.8±2.6	>30	4.3±0.6
**Mutant**			
F74L	13±2.0	>30	4.2±0.8
F116L	6.6±1.3	>30	4.6±0.7
F74, F116L	7.0±0.3	>30	3.7±0.6
R78G	6.7±1.4	>30	2.0±0.4
R78G, F116L	20.0±4.0	>30	8.8±3.3

c

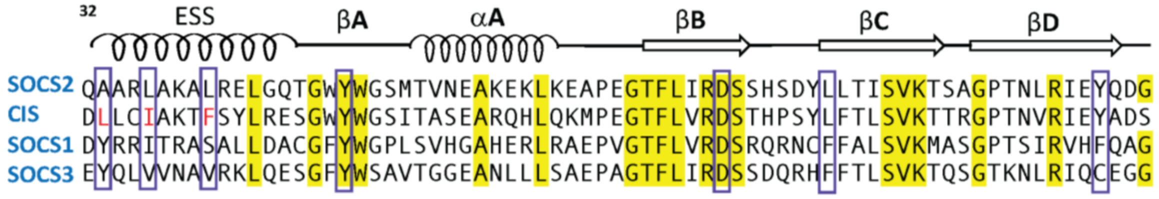

Supplementary Figure 5. Multiple residues in CIS contribute to its reduced affinity for F3. (a) The seven key SOCS2 residues (yellow) which interact with F3 are highlighted on the SOCS2 structure (cyan). The corresponding residues in CIS are shown in pink. (b) Binding affinity of wild-type and mutant CIS proteins for GHR pY595 and F3 peptides, as measured by SPR. Right hand column shows the increase in binding affinity for GHR pY595, when CIS + F3 is present in the cell. Values are the mean and standard deviation of three independent experiments. Source data are provided as a Source Data file. (c) Partial sequence alignment between CIS and SOCS1-3, covering the residues in SOCS2 which interact with F3. Conserved residues are shown in yellow, and the key residues in SOCS2 which contribute to F3 interaction are boxed in purple. Key residues which differ between CIS and SOCS2 are highlighted in red.

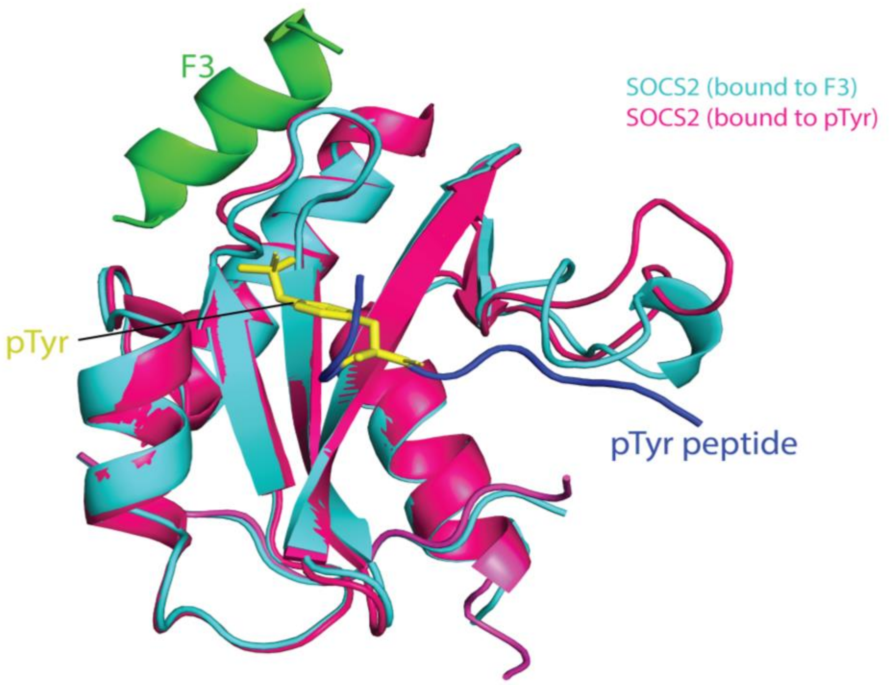

Supplementary Figure 6. Structure alignment of SOCS2 in complex with F3 and pTyr. Structural alignment of SOCS2 (cyan) in complex with F3 (green) and SOCS2 (hot pink) in complex with erythropoietin receptor pTyr peptide (blue; PDB: 6I4X).

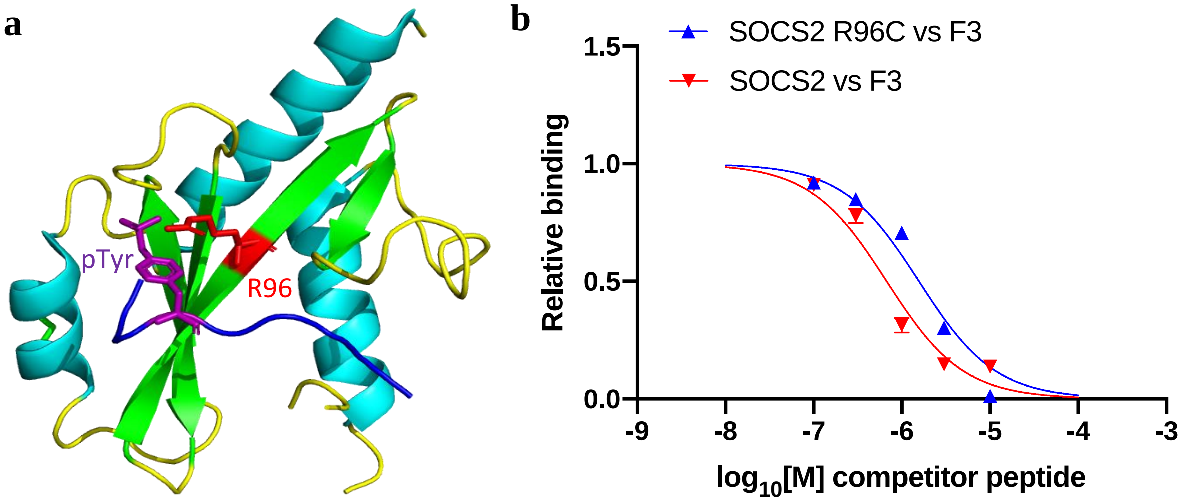

Supplementary Figure 7. Mutation of Arg 96 in the SOCS2-SH2 domain does not impact binding to F3. (a) SOCS2-SH2 domain structure in complex with pTyr peptide (adopted from 6I4X). R96 is highlighted in red. (b) SOCS2-R96C and SOCS2 binding affinities to F3 were tested by SPR, the IC_{50} between SOCS2-R96C and F3 is 1.56±0.33 μM, the IC_{50} between SOCS2 and F3 is 0.67±0.03 μM. Values are mean ± S.D., derived from 3 independent experiments. Source data are provided as a Source Data file.

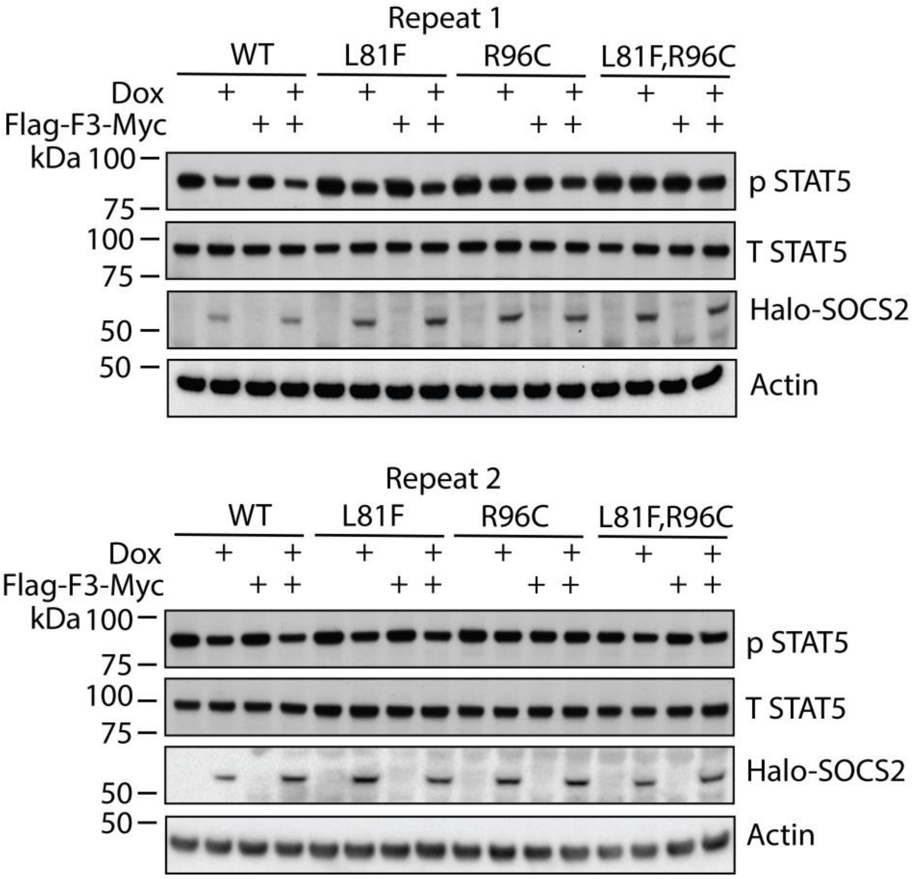

Supplementary Figure 8. Two additional independent replicates demonstrating F3 enhancement of SOCS2 inhibition. A549-GHR cells were transfected with Flag-F3-Myc constructs (F3) prior to overnight doxycycline (dox) treatment to induce wild-type (WT) or mutant Halo-SOCS2 proteins. Cells were then treated with 50 ng/mL GH for 30 min, lysed and analyzed by immunoblotting with antibodies to the indicated proteins. p=phosphorylated; T=total. Images were analyzed by densitometry and included in the compiled data in Figure 5f. Source data are provided as a Source Data file.

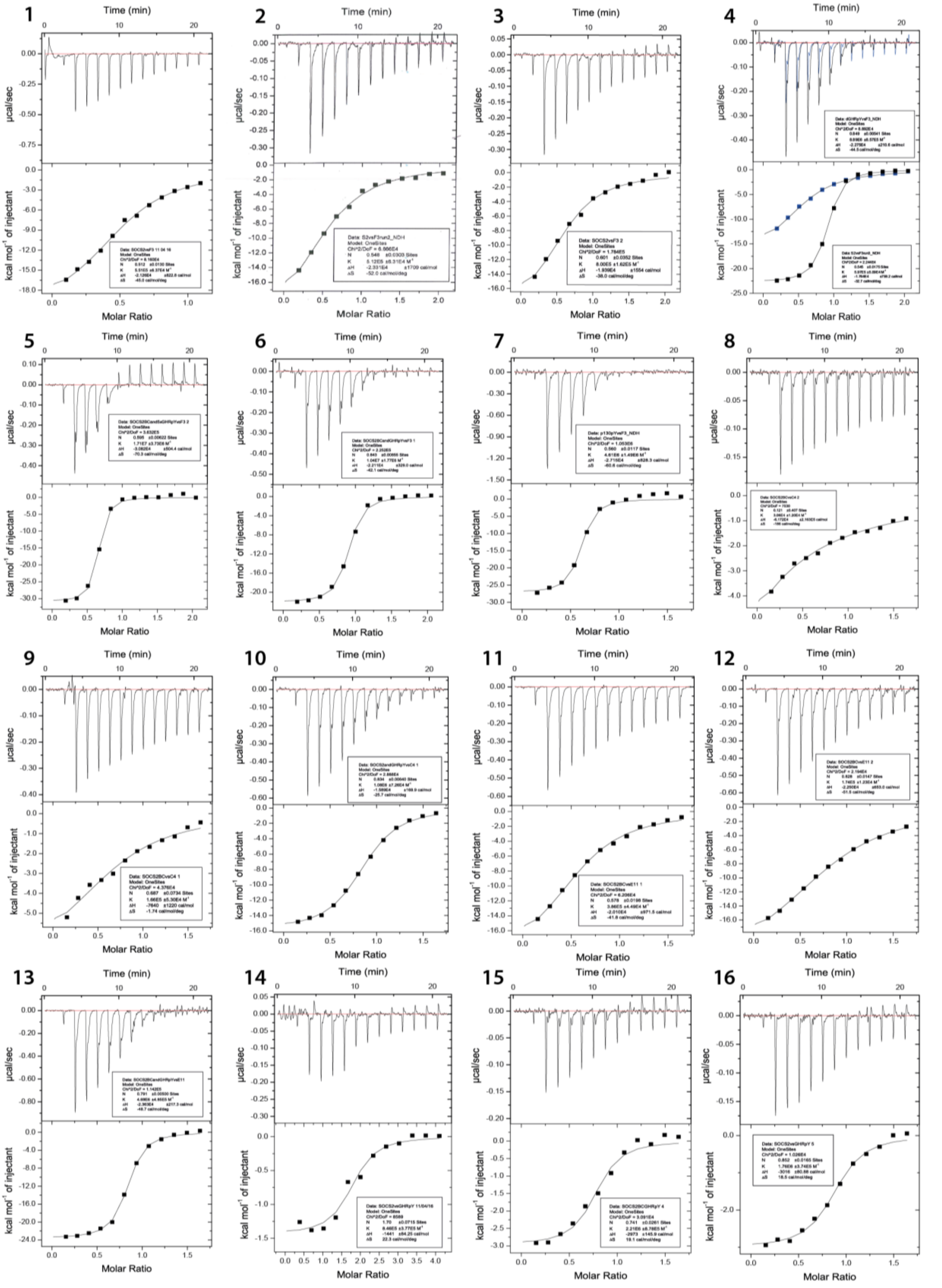
1
2
3
4
5
6
7
8
9
10
11
12
13
14
15
16
Time (min)
µcal/sec
kcal mol⁻¹ of injectant
Molar Ratio

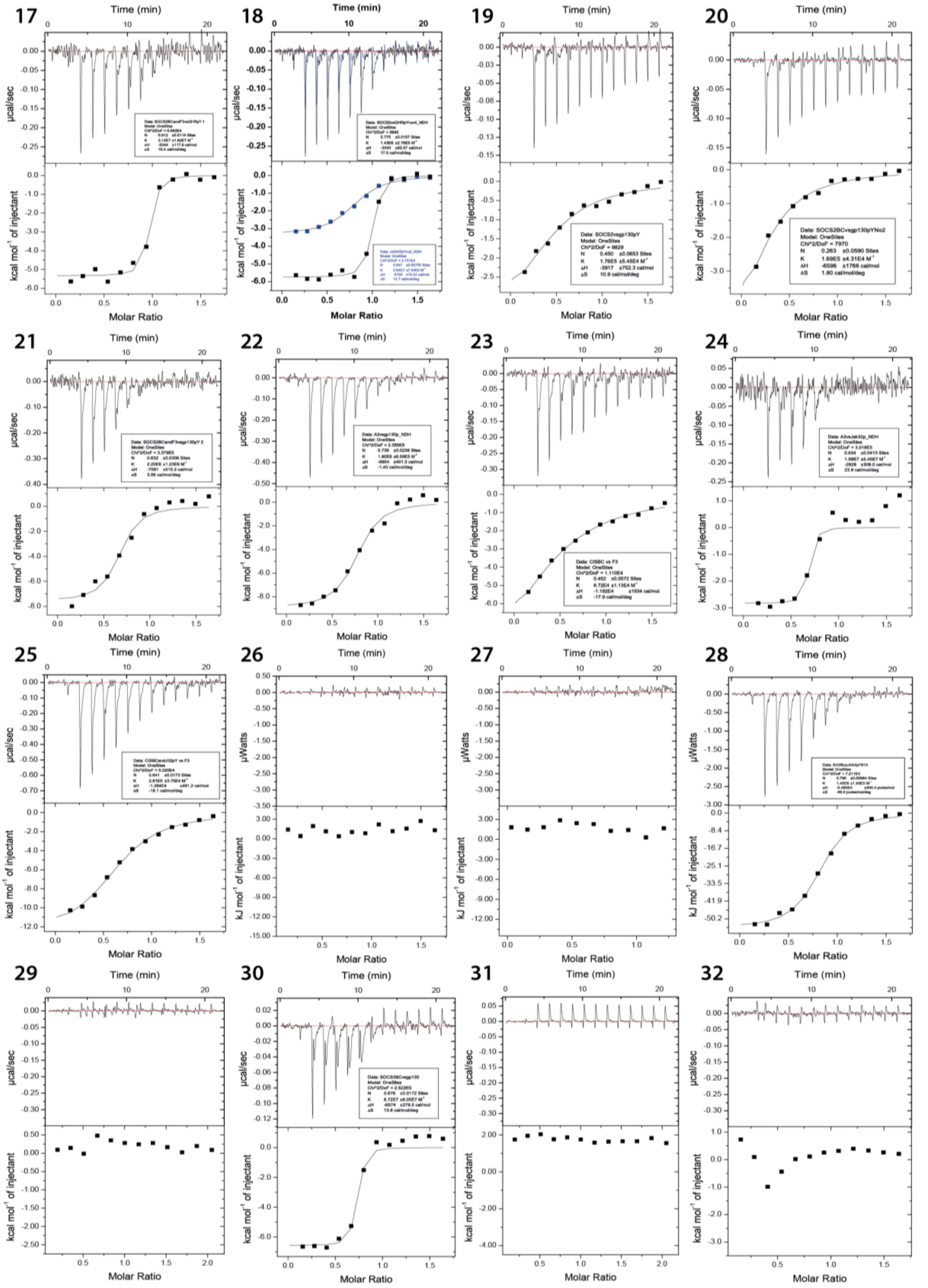
17
18
19
20
21
22
23
24
25
26
27
28
29
30
31
32
Time (min)
Molar Ratio
µcal/sec
kcal mol-1 of injectant
µWatts
kJ mol-1 of injectant

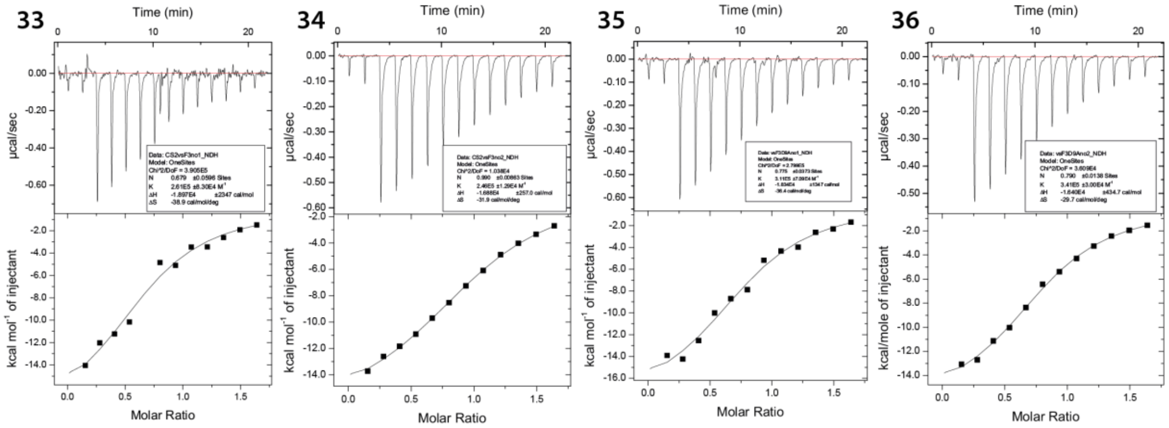

Supplementary Figure 9. ITC curves. Averaged data and individual replicate data are shown in Supplementary Tables 1 & 2. Note that the overlay shown in #18 corresponds to Fig. 2a and the overlay in #4 corresponds to Fig. 2b. Overlay in #4 (SOCS2 + GHR pY595 vs F3) is also shown separately in #6.

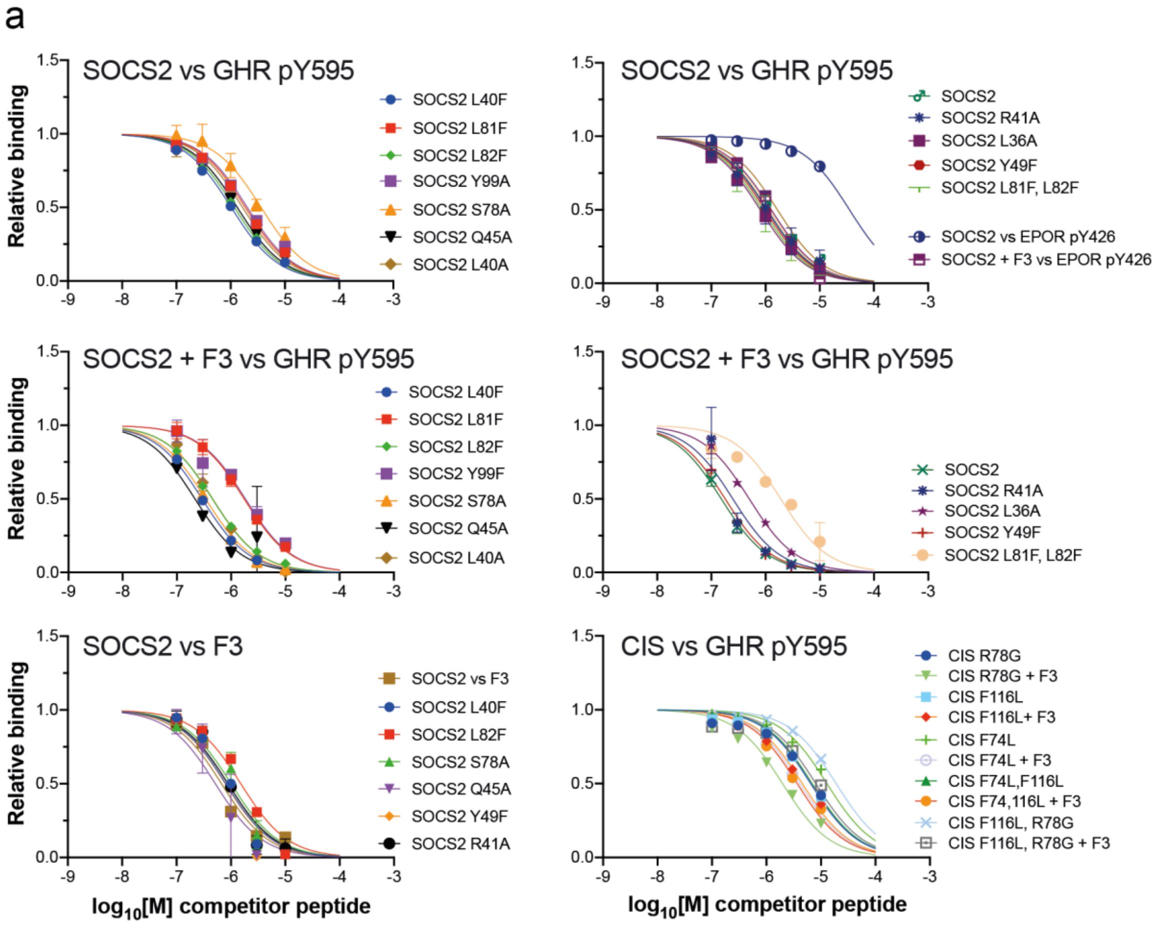

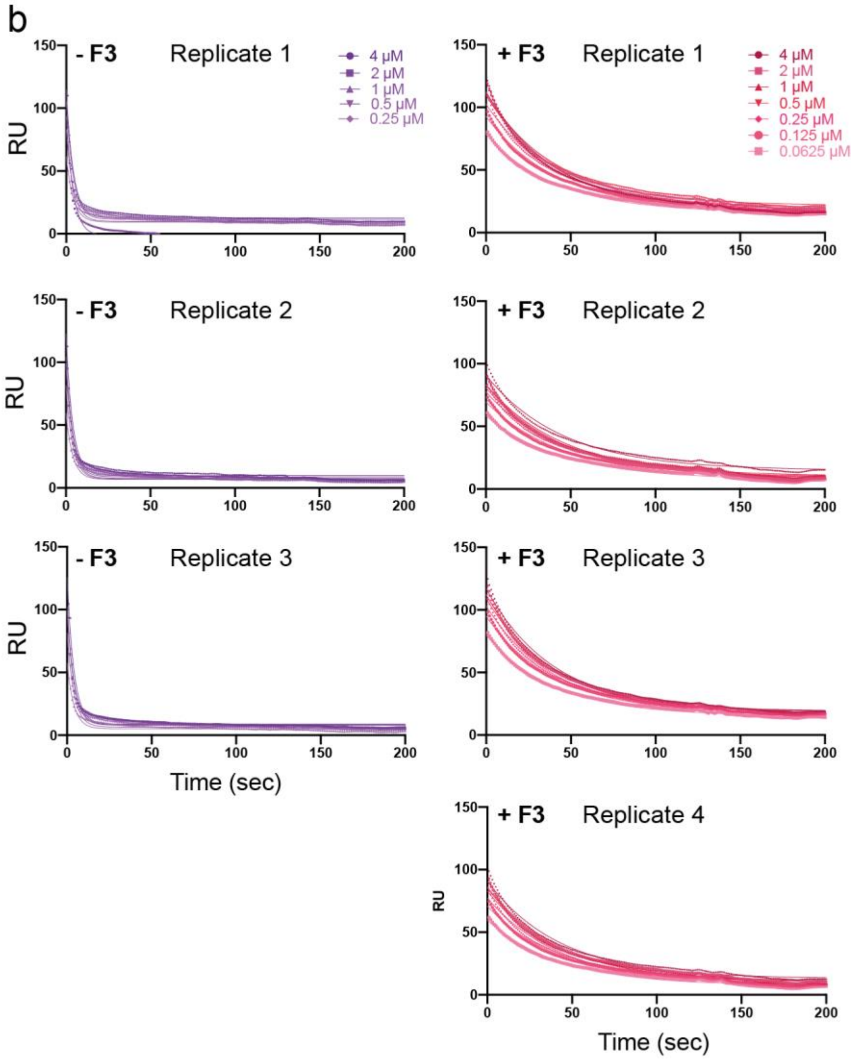

Supplementary Figure 10. Representative SPR curves for competition SPR assays and association/dissociation experiments (a) Representative SPR curves corresponding to data in Tables 1 & 2 and Supplementary Table 1. Values are mean ± S.D. and are derived from n=3 independent experiments. (b) On and off rates in the presence and absence of F3. SPR with a SOCS2 titration was used to generate GHR pY595 peptide association (left panels) and dissociation (right panels) curves. Absolute response (RU) was collected every second, with each data point shown as a dot. Fitted curves as shown as solid lines. One phase association and dissociation analyses with shared Kd across different SOCS2 titrations were performed in GraphPad Prism to calculate k_{off} (dissociation rate): 0.245±0.003 sec^{-1} (-F3) and 0.0235±0.001 sec^{-1} (+F3). Fits for k_{on} values are not provided as they were not sufficiently robust. RU: Response units. Independent replicate experiments. Associated with Fig. 4c. Source data are provided as a Source Data file.

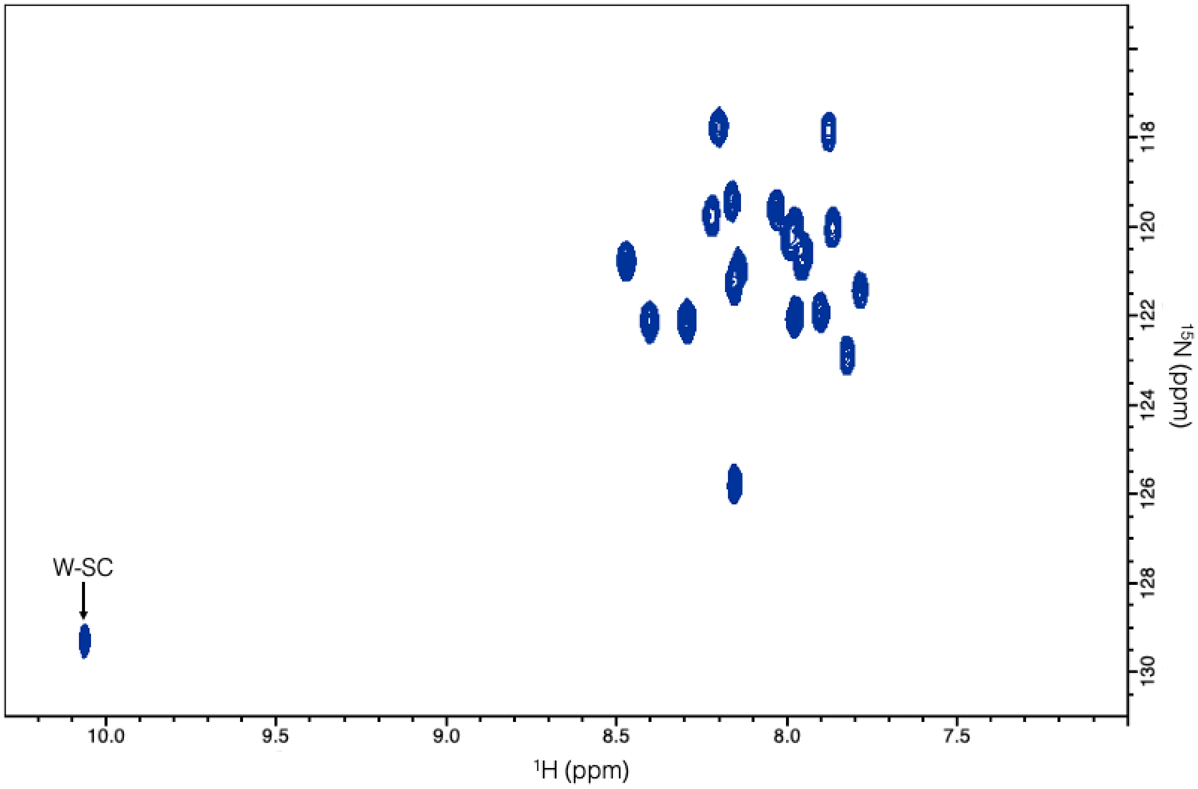

Supplementary Figure 11. ^{1}H-^{15}N HSQC of ^{15}N-labeled F3 peptide. Amide peaks of F3 display random-coil chemical shifts. Resonance at 10 ppm is a tryptophan sidechain (W-SC). NMR spectra were recorded on a 600 MHz Bruker Avance spectrometer equipped with a triple-resonance cryoprobe.

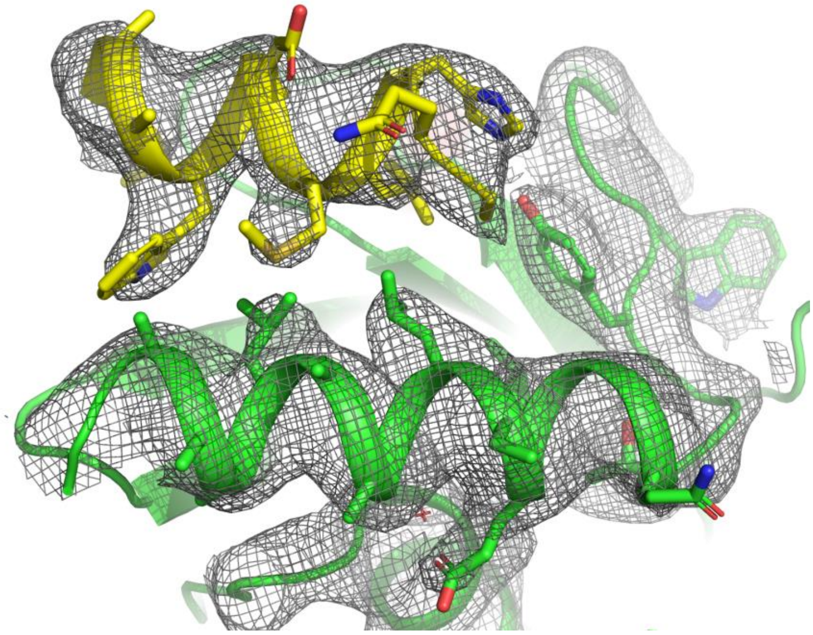

Supplementary Figure 12. 2mFo-DFc map, contoured at 1.0σ, showing the interface of F3 (yellow) with SOCS2 (green).

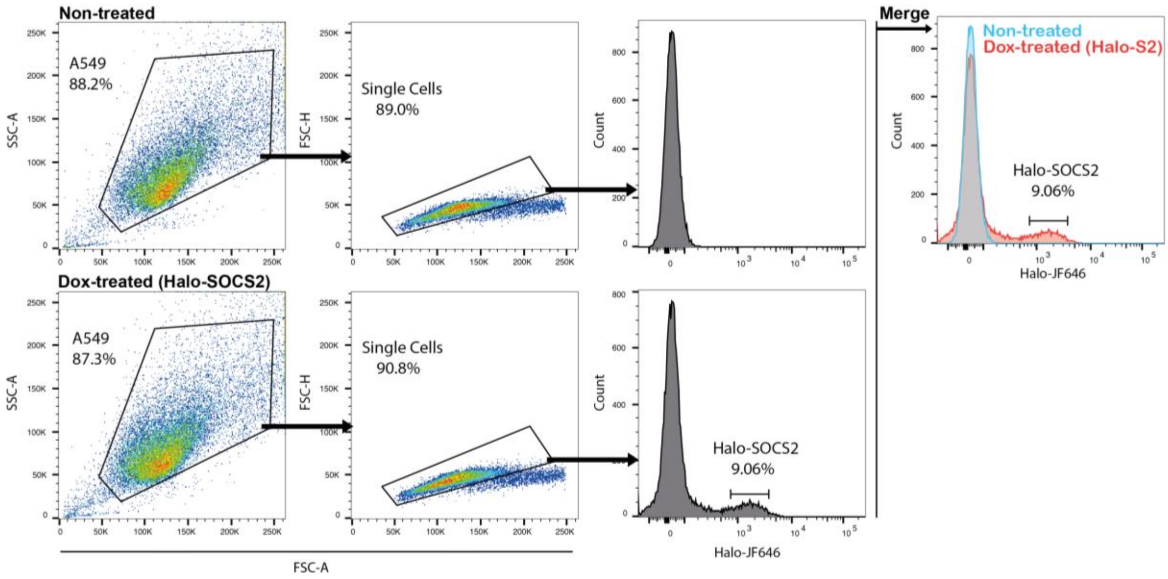

Supplementary Figure 13. Example of gating strategy used to FACS sort A549 cells expressing Halo-SOCS2. Expression of Halo-SOCS2 and SOCS2 mutants was induced with 1 µg/mL doxycycline overnight, together with 10 nM JF646 HaloTag to label Halo-SOCS2 proteins. Cells were initially gated on SSC vs FSC, followed by gating on FSC-H vs FSC-A to exclude doublets. Halo-SOCS2 (JF646 +ve) cells were then selected with the same gate applied to sort SOCS2 wild-type and SOCS2 mutant cells with comparable Halo expression.

Supplementary Table 1. F3 binding is specific to the SOCS2-SH2 domain and can enhance SOCS2-SH2 binding to multiple phosphorylated peptides.

Cell (protein + peptide)	Syringe	Kd (μM)	n
SOCS2-Elo B/C	F3	1.68±0.31	4
SOCS2-Elo B/C + GHRpY595	F3	0.08±0.04	2
SOCS2-Elo B/C + gp130 pY757	F3	0.22	1
SOCS2-Elo B/C	C4	19.33±26.62	2
SOCS2-Elo B/C + GHRpY595	C4	0.93	1
SOCS2-Elo B/C	E11	4.17±3.16	2
SOCS2-Elo B/C + GHRpY595	E11	0.21	1
SOCS2-Elo B/C	GHR pY595	0.73±0.32	4
SOCS2-Elo B/C + F3	GHR pY595	0.04±0.01	2
SOCS2-Elo B/C	gp130 pY757	5.80±0.24	2
SOCS2-Elo B/C + F3	gp130 pY757	0.51±0.11	2
SOCS2-Elo B/C*	EPOR pY426	36.2±5.6*	3
SOCS2-Elo B/C + F3*	EPOR pY426	1.3±0.2*	3
CIS-Elo B/C	F3	14.88	1
CIS-Elo B/C	JAK3 pY980/981	0.8	1
CIS-Elo B/C + F3	JAK3 pY980/981	0.05	1
CIS-Elo B/C + JAK3 pY980/981	F3	3.56	1
SH2B	JAK2 pY813	0.69	1
SH2B	F3	-	1
SOCS3-Elo B/C	gp130 pY757	0.01	1
SOCS3-Elo B/C	F3	NB	1
SOCS2-SB only -Elo B/C	F3	NB	1
No protein + GHR pY595	F3	NB	1

ITC and SPR data showing (i) SOCS2 binding data for the homologous C4 and E11 peptides. (ii) F3 reciprocally enhances SOCS2-SH2 binding to phosphopeptides derived from the IL-6 signaling subunit, gp130 (Y757) and erythropoietin receptor, EPOR (Y426). (iii) F3 binding is specific to the SOCS2-SH2 domain, binding with weaker affinity to the CIS-SH2 domain and not at all to the SOCS3 or SH2B SH2 domains. (iv) F3 binding to CIS similarly enhances binding to a phosphopeptide derived from the JAK3 activation loop. NB: no binding. Where $n \geq 3$ independent experiments, values shown are mean ± S.D; where n=2 independent experiments, values are averages ± range. *IC_{50} measured by SPR. Representative SPR curves are shown in Supplementary Fig. 10a.

Supplementary Table 2. ITC data corresponding to Supplementary Table 1 and including data from replicate experiments.

ITC	Cell (protein + peptide)	Syringe	Ka	Error	ΔH (kcal/mol)	ΔS (cal/mol/deg)	N	Chi^2/Dof	Kd (μM)	+/- error (μM)
1	SOCS2-Elo B/C	F3	5.51E+05	6.37E+04	-2.13E+04	-45.0	0.51	8.16E+04	1.82	0.21
2	SOCS2-Elo B/C	F3	5.12E+05	6.31E+04	-2.33E+04	-52.0	0.55	6.67E+04	1.95	0.24
3	SOCS2-Elo B/C	F3	8.00E+05	1.62E+05	-1.94E+04	-38.0	0.60	1.78E+05	1.25	0.25
4	SOCS2-Elo B/C	F3	5.97E+05	5.09E+04	-1.76E+04	-32.7	0.55	2.24 E+04	1.69	0.22
5	SOCS2-Elo B/C + GHR pY595	F3	1.71E+07	3.73E+06	-3.08E+04	-70.3	0.595	3.63E+05	0.06	0.01
6	SOCS2-Elo B/C + GHR pY595	F3	1.04E+07	1.77E+06	-2.21E+04	-42.1	0.843	2.25E+05	0.10	0.02
7	SOCS2-Elo B/C + gp130 pY757	F3	4.61E+06	1.49E+06	-2.72E+04	-60.6	0.56	1.05E+06	0.22	0.07
8	SOCS2-Elo B/C	C4	3.06E+04	1.20E+04	-6.17E+04	-186	0.121	7.03E+03	32.64	12.78
9	SOCS2-Elo B/C	C4	1.66E+05	5.30E+04	-7.64E+03	-1.74	0.687	4.38E+04	6.02	1.92
10	SOCS2-Elo B/C + GHR pY595	C4	1.08E+06	7.26E+04	-1.59E+04	-25.7	0.834	2.89E+04	0.93	0.06
11	SOCS2-Elo B/C	E11	3.86E+05	4.49E+04	-2.01E+04	-41.8	0.578	6.21E+04	2.59	0.30
12	SOCS2-Elo B/C	E11	1.74E+05	1.23E+04	-2.25E+04	-51.5	0.828	2.19E+04	5.75	0.41
13	SOCS2-Elo B/C + GHR pY595	E11	4.69E+06	4.85E+05	-2.36E+04	-48.7	0.791	1.14E+05	0.21	0.02
14	SOCS2-Elo B/C	GHR pY595	8.46E+05	3.77E+05	-1.44E+03	22.3	1.70	8.59E+03	1.18	0.53
15	SOCS2-Elo B/C	GHR pY595	2.21E+06	8.78E+05	-2.97E+03	19.1	0.741	3.09E+04	0.45	0.18
16	SOCS2-Elo B/C	GHR pY595	1.76E+06	3.74E+05	-3.02E+03	18.5	0.852	1.03E+04	0.57	0.12
17	SOCS2-Elo B/C + F3	GHR pY595	3.13E+07	1.62E+07	-5.34E+03	16.4	0.912	6.68E+04	0.03	0.02
18	SOCS2-Elo B/C	GHR pY595	1.43E+06	2.76E+05	3.34E+03	17	0.775	9.94E+03	0.70	0.14
18	SOCS2-Elo B/C + F3	GHR pY595	2.64E+07	7.54E+06	-5.75E+03	14.7	0.947	2.73E+04	0.04	0.01
19	SOCS2-Elo B/C	gp130 pY757	1.76E+05	5.54E+04	-3.92E+03	10.9	0.45	9.83E+03	5.68	1.79
20	SOCS2-Elo B/C	gp130 pY757	1.69E+05	4.31E+04	-6.60E+03	1.8	0.263	7.97E+03	5.92	1.51

**21**	SOCS2-Elo B/C + F3	gp130 pY757	2.20E+06	1.23E+06	-7.59E+03	3.56	0.632	3.38E+05	0.45	0.25
**22**	SOCS2-Elo B/C + F3	gp130 pY757	1.80E+06	6.59E+05	-8.95E+03	-1.4	0.738	2.36E+05	0.56	0.20
**23**	CIS-Elo B/C	F3	6.72E+04	1.13E+04	-1.19E+04	-17.9	0.452	1.11E+04	14.88	2.50
**24**	CIS-Elo B/C + F3	JAK3 pY980/981	1.99E+07	5.45E+07	-2.83E+03	23.9	0.634	3.02E+05	0.05	0.14
**25**	CIS-Elo B/C + JAK3 pY980/981	F3	2.81E+05	3.75E+04	-1.28E+04	-18.1	0.641	5.02E+04	3.56	0.47
**26**	SH2B	E11	-	-	-	-	-	-	-	-
**27**	SH2B	F3	-	-	-	-	-	-	-	-
**28**	SH2B	JAK2 pY813	1.45E+06	1.93E+05	-5.49E+04	-66	0.79	7.21E+04	0.69	0.09
**29**	SOCS3 B/C	F3	-	-	-	-	-	-	-	-
**30**	SOCS3 B/C	gp130 pY757	6.72E+07	6.05E+07	-6.57E+03	13.8	0.676	2.52E+05	0.015	0.013
**31**	No protein + GHR pY595	F3	-	-	-	-	-	-	-	-
**32**	SOCS2-SB only-Elo B/C	F3	-	-	-	-	-	-	-	
**33**	SOCS2-Elo B/C*	F3	2.61E+05	8.30E+04	-1.90E+04	-38.9	0.68	3.91E+05	4.30	1.40
**34**	SOCS2-Elo B/C*	F3	2.46E+05	1.29E+04	-1.69E+04	-31.9	0.99	1.04E+04	4.08	0.22
**35**	SOCS2-Elo B/C*	F3 D9A	3.11E+05	7.09E+04	-1.83E+04	-36.4	0.78	2.80E+05	3.39	0.77
**36**	SOCS2-Elo B/C*	F3 D9A	3.41E+05	3.00E+04	-1.64E+04	-29.7	0.79	3.61E+04	2.96	0.26
**SuppFig1**	SOCS2-Elo B/C	F3	3.55E+05	4.37E+04	-1.66E+04	-30.4	0.81	5.18E+04	2.8	0.30
**SuppFig1**	SOCS2-Elo B/C+Cullin5 NTD	F3	4.77E+05	2.34E+04	-1.70E+04	-31.0	1.02	1.12E+04	2.1	0.10
**SuppFig1**	SOCS2-Elo B/C	Cullin5 NTD	5.17E+07	2.67E+07	-4.56E+03	20	0.83	3.09E+04	0.026	0.013
**SuppFig1**	SOCS2-Elo B/C + F3	Cullin5 NTD	2.39E+07	8.89E+06	-5.43E+03	15.6	0.81	4.17E+04	0.049	0.018

*ITC curves #33-36 were performed within one experiment, with the same protein batch. Note the Kd for F3 differs slightly from other experiments.

Supplementary Table 3. Peptides with identity to F3 tested by SPR for interaction with SOCS2

**Protein (*Homo sapiens*)**	**Peptide sequences**
MIA2*	EKSKLLEKFSLV
ARMD1*	ARSHILDKFIET
MABP1*	SDRRLLDKWVEL
OAS*	TPGHLLDKFIKE
CFA54*	GQPHLLNKFNFV
MYCBP2*	GIKKAALMHKWPLKEI
MYCBP2*	SMMCPPGMHKWKLEQC
F3	DIALQHLMDKWMAMQN

*None of the peptides displayed detectable binding.
Residues that are identical in F3 are highlighted in red.

Supplementary Table 4. DNA construct list

Construct	Species	Residues	Vector
***E coli* expression constructs**			
GST- CIS	Human	ΔN-terminal, ΔPEST 35-173, 202-258	pGEX-4T
GST- CIS mutations	Human	ΔN-terminal, ΔPEST 35-173, 202-258	pGEX-4T
GST- SOCS2 FL	Human	1-198	pGEX-4T
GST- SOCS2	Human	32-198	pGEX-4T
GST- SOCS2 mutations	Human	32-198	pGEX-4T
GST-SOCS2 (SH2 only)	Human	32-159	pGEX-4T
GST- SOCS2 KKQ	Human	ΔN-terminal, K115A/K117A/Q118A	pGEX-4T
Elongin B and Elongin C	Human	Elongin B 1-118, Elongin C 17–112	pACYCDUET
GST-SOCS2 SOCS-box	Human	154-198	pGEX-4T
(6XHis)-SH2B	Human	1-114	PET-15
GST-Cullin5	Human	1-485	pGEX-4T
**Mammalian expression constructs**			
FLAG-SOCS2	Human	1-198	pEF-BOS
FLAG-SOCS2 mutation	Human	1-198	pEF-BOS
Halo-SOCS2	Human	1-198	Tre3g
Halo-SOCS2 mutation	Human	1-198	Tre3g
GST-F3-Myc	-	-	pEF-BOS
Flag-F3-Myc	-	-	pEF-BOS
GST-Myc	-	-	pEF-BOS
mRuby3-F3-Myc	-	-	PMSCV
mRuby3-Myc	-	-	PMSCV
HA-GHR	Human	1-638	pQCXP
pMDL (lenti packaging vector)			Tre3g
RSV-REV (lenti packaging vector)			Tre3g
VSVg (lenti envelope vector)			Tre3g

Supplementary Table 5. Data collection and refinement statistics

	SOCS2/EloB/EloC/F3
Wavelength	0.9537
Resolution range	43.383 - 3.194 (3.308 - 3.194)
Space group	C 2 2 21
Unit cell	60.749 110.612 139.896 90 90 90
Total reflections	53284 (4745)
Unique reflections	8106 (780)
Multiplicity	6.6 (6.1)
Completeness (%)	99.29 (95.86)
Mean I/sigma(I)	14.20 (1.62)
Wilson B-factor	104.31
R-merge	0.09654 (1.157)
R-meas	0.105 (1.265)
R-pim	0.04074 (0.5039)
CC1/2	0.999 (0.782)
CC*	1 (0.937)
Reflections used in refinement	8074 (764)
Reflections used for R-free	807 (69)
R-work	0.2271 (0.4054)
R-free	0.2667 (0.5577)
CC(work)	0.942 (0.791)
CC(free)	0.932 (0.514)
Number of non-hydrogen atoms	2670
macromolecules	2646
ligands	23
solvent	1
Protein residues	350
RMS(bonds)	0.003
RMS(angles)	0.87
Ramachandran favored (%)	96.05
Ramachandran allowed (%)	3.04
Ramachandran outliers (%)	0.91
Rotamer outliers (%)	2.62
Clashscore	2.69
Average B-factor	110.40
macromolecules	110.17
ligands	137.72
solvent	100.27

Statistics for the highest-resolution shell are shown in parentheses.

Supplementary Table 6. List of primers used for site-directed mutagenesis and sequencing

Primers	Sequences
SOCS2 L36A	caggcggcgcgtgcggcgaaggccctg
SOCS2 L40F	tctggcgaaggccttccgggagctcggtc
SOCS2 L40A	ctggcgaaggccgcgcgggagctcggt
SOCS2 R41A	ggcgaaggccctggcggagctcggtcag
SOCS2 Q45A	ctgcgggagctcggtgcgacaggatggtactg
SOCS2 Y49F	ggtcagacaggatggttctggggaagtatgact
SOCS2 S78A	cttgattagagatagctcgcatgcagactacctactaacaatat
SOCS2 Y99A	ctaatcttcgaatcgaagcccaagacggaaaattcagat
SOCS2 L81F	tagctcgcattcagactacttcctaacaatatctgttaa
SOCS2 L82F	atagctcgcattcagactacctattcacaatatctgttaa
SOCS2 L81, L82F	atagctcgcattcagactacttcttcacaatatctgttaaaacat
SOCS2 R96C	ccgtcttggtattcgatgcaaagattagttggtccagctgatg
CIS F74L	cgaaggtaggataaggtcttggctatgcacagc
CIS F116L	gccagattccccaaggtaggagaaggtcttg
CIS R78L	ctgacagcgttaacaggtagctggggtgc
pGEX-5’ sequencing primer	ggctggcaagccacgtttggtg
pGEX-3’ sequencing primer	cgggagctgcatgtgtcagagg
pEFBOS-5’ sequencing primer	caagcctcagacagtggttca
pEFBOS-3’ sequencing primer	cagggatttcttgtctcccacg
