# Supplementary material for: Discovery of an exosite on the SOCS2-SH2 domain that enhances SH2 binding to phosphorylated ligands
Source: Nat Commun. 2021 Dec 2;12:7032. doi: 10.1038/s41467-021-26983-5 (PMC8640019; doi:10.1038/s41467-021-26983-5)
Supplement: Supplementary file 3 — Reporting Summary [file 41467_2021_26983_MOESM3_ESM.pdf]

nature research

Corresponding author(s): Sandra Nicholson and Nadia Kershaw

Last updated by author(s): Oct 2, 2021

Reporting Summary

Nature Research wishes to improve the reproducibility of the work that we publish. This form provides structure for consistency and transparency in reporting. For further information on Nature Research policies, see our Editorial Policies and the Editorial Policy Checklist.

Statistics

For all statistical analyses, confirm that the following items are present in the figure legend, table legend, main text, or Methods section.

n/a	Confirmed	
[ ]	[x]	The exact sample size (*n*) for each experimental group/condition, given as a discrete number and unit of measurement
[ ]	[x]	A statement on whether measurements were taken from distinct samples or whether the same sample was measured repeatedly
[ ]	[x]	The statistical test(s) used AND whether they are one- or two-sided<br>*Only common tests should be described solely by name; describe more complex techniques in the Methods section.*
[x]	[ ]	A description of all covariates tested
[x]	[ ]	A description of any assumptions or corrections, such as tests of normality and adjustment for multiple comparisons
[ ]	[x]	A full description of the statistical parameters including central tendency (e.g. means) or other basic estimates (e.g. regression coefficient) AND variation (e.g. standard deviation) or associated estimates of uncertainty (e.g. confidence intervals)
[ ]	[x]	For null hypothesis testing, the test statistic (e.g. *F*, *t*, *r*) with confidence intervals, effect sizes, degrees of freedom and *P* value noted<br>*Give P values as exact values whenever suitable.*
[x]	[ ]	For Bayesian analysis, information on the choice of priors and Markov chain Monte Carlo settings
[x]	[ ]	For hierarchical and complex designs, identification of the appropriate level for tests and full reporting of outcomes
[x]	[ ]	Estimates of effect sizes (e.g. Cohen's *d*, Pearson's *r*), indicating how they were calculated

Our web collection on statistics for biologists contains articles on many of the points above.

Software and code

Policy information about availability of computer code

Data collection	ITC data were collected using a Microcal ITC200.<br>TSA fluorescent data were acquired on a CFX384 Real-Time System (Biorad).<br>SPR data were acquired on a BIACORE 4000.<br>NMR spectra were acquired on a Bruker Avance 600 MHz spectrometer.<br>X-ray data were collected at the Australian Synchrotron.<br>FACS data were collected using Cell quest software (BD Biosciences) on a BD FACSAria III cell sorter.
Data analysis	ITC data were analyzed using Microcal Origin 7.0.<br>TSA data were analyzed using Prism 9.0.0.<br>SPR data were analyzed using Biacore Evaluation software and Prism 8 (version 8.0.2).<br>SOCS2 (-/+F3) dissociation rates were analyzed with Prism 9 (version 9.0.0) using model "Dissociation-One phase exponential decay".<br>Fluorescence lifetime data were analysed using FLIMfit (v4.12.149). Mono-exponential fluorescence lifetimes were fitted pixel-wise (11x11 bin) for the FLIM images, and fitted image-wise for the plots and statistical analysis.<br>X-ray data were integrated using XDS and structure determined using PHASER, PHENIX and COOT.<br>NMR spectra were processed in TopSpin and analyzed in CARA.<br>FACS data were analyzed using FlowJo 10 software.

For manuscripts utilizing custom algorithms or software that are central to the research but not yet described in published literature, software must be made available to editors and reviewers. We strongly encourage code deposition in a community repository (e.g. GitHub). See the Nature Research guidelines for submitting code & software for further information.

Data

Policy information about availability of data

All manuscripts must include a data availability statement. This statement should provide the following information, where applicable:

- Accession codes, unique identifiers, or web links for publicly available datasets
- A list of figures that have associated raw data
- A description of any restrictions on data availability

The coordinates and structure factors of human SOCS2 in complex with elongins B and C and F3 peptide generated in this study have been deposited in the RSCB Protein Data Bank (PDB) under accession code 7M6T.
The NMR assignments generated in this study have been deposited with the Biological Magnetic Resonance Bank under accession codes 50868 (SOCS2-SH2 bound to GHRpY peptide), and 50869 (SOCS2-SH2 bound to GHRpY peptide and F3 peptide). Previously published crystal structures used in this study are available from the PDB under accession codes 2C9W, 5BO4 and 6I4X. Source data are provided with this paper as Source Data file-SPR raw data and Source Data file-uncropped western blots.

Field-specific reporting

Please select the one below that is the best fit for your research. If you are not sure, read the appropriate sections before making your selection.

- [x] Life sciences
- [ ] Behavioural & social sciences
- [ ] Ecological, evolutionary & environmental sciences

For a reference copy of the document with all sections, see nature.com/documents/nr-reporting-summary-flat.pdf

Life sciences study design

All studies must disclose on these points even when the disclosure is negative.

Sample size	Statistical analysis was not used to determine sample size, but experiments were repeated 2-3 times independently to verify the results.
Data exclusions	Data was excluded where there was technical error (e.g. poor transfection efficiency, poor or unequal protein transfer in immunoblotting).
Replication	Experiments were repeated independently in order to ascertain reproducibility and the number of repeats performed for data presented in each figure panel is clearly stated in the figure legends. In cases where variability was observed more repeats were generated, unless there was obvious technical error.
Randomization	The nature of the in vitro experiments, with technical replicates and internal comparisons, did not require randomization of samples for analysis.
Blinding	Blinding was not necessary for in vitro experiments as within each experiment, all samples were analyzed simultaneously using identical assay conditions and reagents.

Reporting for specific materials, systems and methods

We require information from authors about some types of materials, experimental systems and methods used in many studies. Here, indicate whether each material, system or method listed is relevant to your study. If you are not sure if a list item applies to your research, read the appropriate section before selecting a response.

Materials & experimental systems

n/a	Involved in the study	
[ ]	[x]	Antibodies
[ ]	[x]	Eukaryotic cell lines
[x]	[ ]	Palaeontology and archaeology
[x]	[ ]	Animals and other organisms
[x]	[ ]	Human research participants
[x]	[ ]	Clinical data
[x]	[ ]	Dual use research of concern

Methods

n/a	Involved in the study	
[x]	[ ]	ChIP-seq
[ ]	[x]	Flow cytometry
[x]	[ ]	MRI-based neuroimaging

Antibodies

Antibodies used	Anti-phosphotyrosine antibody (4G10) (Sigma-Aldrich; ab179530; 1:2000).<br>Anti-STAT3 antibody (79D7) (Cell Signaling; #4904; 1:2000)<br>Anti-Myc antibody (71D10) (Cell Signaling; #3946, 1:2000)<br>Anti-STAT5 antibody (D2O6Y) (Cell Signaling; #94205; 1:3000)<br>Anti-phospho-STAT5 antibody (C11C5) (Cell Signaling; #9359; 1:2000)

	Anti-phospho-JAK2 antibody (Millipore; 07-606; 1:1000)<br>Anti-JAK2 antibody (Santa Cruz; sc-390539; 1:1000)<br>Anti-Halo antibody (Promega; G921A; 1:2000)<br>Anti-HA antibody (3F10) (Sigma Aldrich; 12158167001; 1:1000)<br>Anti-actin-HRP antibody (C4) (Santa Cruz; sc-47778 HRP; 1:1000)<br>Rat anti-Flag antibody was a kind gift from Prof. D. Huang & Dr. L. O'Reilly (Walter and Eliza Hall Institute; 1:2500)<br>Horseradish peroxidase-conjugated goat anti-rat immunoglobulin (Southern Biotech; 3010-05; 1:10,000)<br>Horseradish peroxidase-conjugated sheep anti-rabbit immunoglobulin (Southern Biotech; 4010-05; 1:15,000)<br>Horseradish peroxidase-conjugated sheep anti-mouse immunoglobulin (GE Healthcare; NA931-1ML; 1:10,000)
Validation	Anti-phosphotyrosine (4G10) (Millipore; ab179530) validated by the manufacturer [https://www.abcam.com/phosphotyrosine-antibody-epr16871-ab179530.html]<br>Anti-STAT3 (79D7) (Cell Signaling; #4904) validated by the manufacturer [https://www.cellsignal.com/products/primary-antibodies/stat3-79d7-rabbit-mab/4904]<br>Anti-Myc (71D10) (Cell Signaling; #3946) validated by the manufacturer and in-house on Myc-tagged proteins [https://www.cellsignal.com/products/primary-antibodies/myc-tag-71d10-rabbit-mab/2278?site-search-type=Products&N=4294956287&Ntt=anti-myc+antibody+71d10&fromPage=plp]<br>Anti-STAT5 (D2O6Y) (Cell Signaling; #94205) validated by the manufacturer [https://www.cellsignal.com/products/primary-antibodies/stat5-d2o6y-rabbit-mab/94205?site-search-type=Products&N=4294956287&Ntt=anti-total+stat5+%28d206y%29+&fromPage=plp]<br>Anti-phospho-STAT5 (C11C5) (Cell Signaling; #9359) validated by the manufacturer [https://www.cellsignal.com/products/primary-antibodies/phospho-stat5-tyr694-c11c5-rabbit-mab/9359?site-search-type=Products&N=4294956287&Ntt=anti-phospho-stat5+%28c11c5%29+&fromPage=plp&_requestid=61977]<br>Anti-phospho-JAK2 (Millipore; 07-606) validated by the manufacturer [https://www.merckmillipore.com/AU/en/product/Anti-phospho-JAK2-Tyr1007-1008-Antibody,MM_NF-07-606?ReferrerURL=https%3A%2F%2Fwww.google.com%2F&bd=1]<br>Anti-JAK2 (C-10) (Santa Cruz; sc-390539) validated by the manufacturer [https://www.scbt.com/p/jak2-antibody-c-10]<br>Anti-Halo (Promega; G921A) validated in-house on Halo-tagged proteins.<br>Anti-HA (3F10) (Sigma Aldrich; 12158167001) validated in-house on HA-tagged proteins.<br>Anti-actin-HRP (C4) (Santa Cruz; sc-47778 HRP) validated by the manufacturer [https://www.scbt.com/p/beta-actin-antibody-c4?requestFrom=search]<br>Rat anti-Flag antibody (Walter and Eliza Hall Institute) was validated in-house on Flag-tagged proteins.

Eukaryotic cell lines

Policy information about cell lines

Cell line source(s)	Human embryonic kidney 293 cells (ATCC® CRL-3216™) and HepG2 cells (ATCC® HB-8065) were from the ATCC and A549 (ATCC® CCL-185™) was a gift from Professor Philip Hansbro, Centenary Institute, Sydney.
Authentication	None of the cell lines used were authenticated.
Mycoplasma contamination	All cell lines were tested for mycoplasma using PCR-based methods and were negative.
Commonly misidentified lines (See ICLAC register)	No commonly misidentified lines were used.

Flow Cytometry

Plots

Confirm that:

- [x] The axis labels state the marker and fluorochrome used (e.g. CD4-FITC).
- [x] The axis scales are clearly visible. Include numbers along axes only for bottom left plot of group (a 'group' is an analysis of identical markers).
- [x] All plots are contour plots with outliers or pseudocolor plots.
- [x] A numerical value for number of cells or percentage (with statistics) is provided.

Methodology

Sample preparation	Live A549 cells were incubated with 10 nM JF456 in culture media overnight, and washed in PBS containing 2% FBS prior to FACS sorting.
Instrument	A549 cells were sorted using a BD FACSAria III cell sorter.
Software	Data were analyzed using FlowJo 10 software.
Cell population abundance	Cells were sorted as 100% Halo +ve. Population abundance is not relevant.

Gating strategy	A549 cells were initially gated on FSC and SSC to identify a single cell population, and then gated on JF646 +ve (Halo +ve) cells for sorting and expansion in cell culture.

☒ Tick this box to confirm that a figure exemplifying the gating strategy is provided in the Supplementary Information.
